# High Thermal Dissipation of Al Heat Sink When Inserting Ceramic Powders by Ultrasonic Mechanical Coating and Armoring

**DOI:** 10.3390/ma10050454

**Published:** 2017-04-26

**Authors:** Wei-Yu Tsai, Guan-Rong Huang, Kuang-Kuo Wang, Chin-Fu Chen, J. C. Huang

**Affiliations:** 1 Department of Materials and Optoelectronic Science, National Sun Yat-Sen University, Kaohsiung 804, Taiwan; wayne2ferrari@gmail.com (W.-Y.T.); kk_wang@mail.nsysu.edu.tw (K.-K.W.); 2 Physics Division, National Center for Theoretical Sciences, Hsinchu 30013, Taiwan; d01222004@ntu.edu.tw; 3 Metal Industries Research & Development Centre, Kaohsiung 811, Taiwan; chencf@mail.mirdc.org.tw; 4 Institute for Advanced Study, City University of Hong Kong, Hong Kong, China

**Keywords:** mechanical coating, ceramic coating, heat dissipation, thermal properties

## Abstract

Aluminum alloys, which serve as heat sink in light-emitting diode (LED) lighting, are often inherent with a high thermal conductivity, but poor thermal total emissivity. Thus, high emissive coatings on the Al substrate can enhance the thermal dissipation efficiency of radiation. In this study, the ultrasonic mechanical coating and armoring (UMCA) technique was used to insert various ceramic combinations, such as Al_2_O_3_, SiO_2_, or graphite, to enhance thermal dissipation. Analytic models have been established to couple the thermal radiation and convection on the sample surface through heat flow equations. A promising match has been reached between the theoretical predictions and experimental measurements. With the adequate insertion of ceramic powders, the temperature of the Al heat sinks can be lowered by 5–11 °C, which is highly favorable for applications requiring cooling components.

## 1. Introduction

Coatings with a high emissivity, which can advance the overall thermal dissipation property, are widely adopted in many devices to transfer more heat by thermal radiation [1,2,3]. According to early researches [4,5,6,7,8,9,10,11], the light output from a light-emitting diode (LED) decreases with an increasing LED junction temperature, and the heat is generally removed from the device by conduction or convection. Without adequate heat transfer by conduction through heat sink or convection through ventilation, the device temperature will rise, resulting in reduced luminous maintenance and a shortened useful life. Similar situations occur in critical parts of computer devices such as central processing units (CPUs) and graphics processing units (GPUs), where accumulated heat deteriorates the performance and durability. Higher power electronic devices need a larger volume of heat sink to dissipate more heat [12,13,14,15]. As a consequence, there is always an urgent need to improve the efficiency of thermal dissipation by fabricating coatings of a high total emissivity onto devices, without increasing the overall volume.

Many techniques have been established for achieving high emissive coatings, like physical or chemical vapor deposition (PVD or CVD) [16,17,18], as well as high velocity oxy-fuel spraying (HVOF) [19], micro-arc oxidation (MAO) [20], and the sol-gel process [21]. However, restrictions have been encountered, such as the oxidation of the substrate and coated material, poor coat-substrate adherence, and the most important issue, the cost [22,23]. There still remain great challenges to achieve effective and economically competitive processes of low cost.

In this study, a mechanical plating method was designed to produce coatings with a high emissivity on the 1050 commercially pure Al, by adding various ceramic powders (with a high thermal radiation capability) into the surface layer. This method is called ultrasonic mechanical coating and armoring (UMCA), initially proposed by Komarov et al. [24,25], allowing a coating to be applied at room temperature and atmospheric pressure in a relatively short time. UMCA is, in fact, an extended application of the surface mechanical attrition treatment (SMAT) process, which was first proposed by Lu [26,27,28]. The dominant benefit of UMCA may be that it offers a high flexibility for choosing and combining various materials for composite coatings. Coatings with excellent properties can be achieved without any chemical and thermal treatment, and also without any produced waste. After many heating-cooling cycles, the coating can still remain intact.

Through this work, we use the UMCA process for coating materials with a high emissivity to an aluminum-fin heat sink, without using an adhesive like traditional spread coating. Prior to this study, the UMCA process had not been applied on LED devices. This new and simple coating technique allows one to complete a high coverage on the surface of a sample with a complex shape in a very short period of time and with a low cost.

## 2. Model of Heat Loss

**Figure 1:**
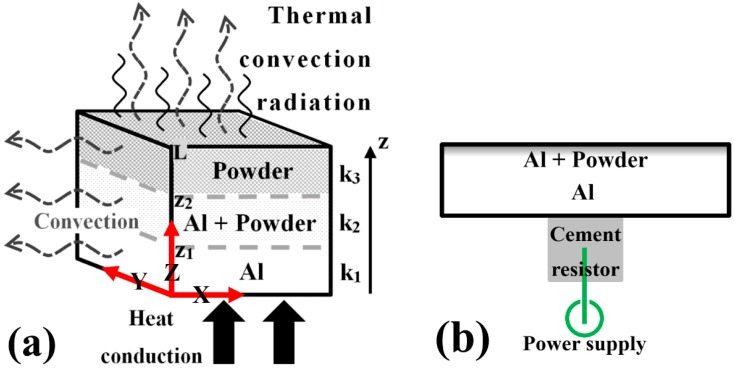
Illustration of (**a**) the concept and (**b**) the experimental setup of heat dissipation.

To verify that thermal radiation is the dominant factor of thermal dissipation efficiency in the experiments, we introduce the model of heat loss. The designed sample configuration, shown in Figure 1, has a constant sample input power from the bottom surface, where the thermal conduction through the sample, and the thermal convection and radiation from the surfaces of the whole sample (particularly from the sample’s top surface), are considered. The *X*, *Y*, and *Z* axes, as adopted below for model deviation, are also defined in Figure 1a.

### 2.1. Thermal Radiation and Convection

Any material with a temperature T above 0 K will emit thermal radiation, and the process is governed by the Stefan-Boltzmann law [29], as follows:

(1) $$P_{r}=\sigma \varepsilon A\left(T^{4}-T_{s}^{4}\right)$$

where $P_{r}$ is the power of thermal radiation, σ is the Stefan-Boltzmann constant, ε is the total emissivity of the material between 0 and 1, A is the surface area of the material, and $T_{s}$ is the surrounding temperature. Furthermore, thermal convection will occur between the sample surface and the air, governed by the equation [30,31,32]:

(2) $$P_{c}=\mathrm{hA}\left(T-T_{s}\right)$$

where $P_{c}$ is the power of thermal convection, h is the convection coefficient or average heat transfer coefficient, and A is the surface area. The dimensions of the cuboid sample used in this paper are $38\times 18\times 5{\;\mathrm{mm}}^{3}$, and the values for the convection heat transfer coefficient, h, can be found in published papers [30,31,32,33,34]. Here, we apply the model of Yovanovich et al. in Ref. [30,31,32], and consider the convection in our experiments to be Laminar natural convection. For the sake of simplicity, h is usually written as a function of the dimensionless Nusselt number Nu in the form of $\mathrm{Nu}={\mathrm{hL}}_{c}/k_{\mathrm{air}}$, where $L_{c}$ is the characteristic length of the test samples (or the cement resistor). For the cuboid test samples, $L_{c}$ is the square root of the wetted sample surface in air, and $k_{\mathrm{air}}$ is the thermal conductivity of air. In general cuboid cases with a dimension of a×b×c [30,31,32], the Nu is related to the Rayleigh number $R_{a}$ in the form:

(3) $$\mathrm{Nu}={\mathrm{Nu}}_{0}+F\left(P_{\mathrm{ran}.}\right)\times G\times R_{a}^{1/4}$$

where the diffusive limit of a cuboid is:

(4) $${\mathrm{Nu}}_{0}=\left[3.192+1.868{\left(a/c\right)}^{0.76}\right]/{\left[1+1.189\left(a/c\right)\right]}^{1/2}$$

the universal Prandtl number function is:

(5) $$F\left(P_{\mathrm{ran}.}\right)=0.670/{\left[1+{\left(0.5/P_{\mathrm{ran}.}\right)}^{9/16}\right]}^{4/9}$$

the body gravity function of a cuboid is:

(6) $$G=2^{1/8}{\left[\frac{0.625a^{4/3}\;b+c{\left(a+b\right)}^{4/3}}{{\left(\mathrm{ab}+\mathrm{ac}+\mathrm{bc}\right)}^{7/6}}\right]}^{3/4}$$

$P_{\mathrm{ran}.}$ is the Prandtl number, and:

(7) $$R_{a}=g\beta \left(T-T_{s}\right)L_{c}^{3}{\left(v\alpha \right)}^{-1}=C\left(T-T_{s}\right),C={g\beta L}_{c}^{3}{\left(v\alpha \right)}^{-1}$$

in which g is the gravitational acceleration, β is the thermal expansion coefficient of air, v is the kinematic viscosity of air, and α is the thermal diffusivity of air. As a consequence, h can be expressed as the following formula:

(8) $$h=h_{0}+h_{c}{\left(T-T_{s}\right)}^{1/4}$$

where $h_{0}$ and $h_{c}$ are:

(9) $$h_{0}=\frac{k_{\mathrm{air}}}{L_{c}}{\mathrm{Nu}}_{0},{\;h}_{c}=\frac{k_{\mathrm{air}}}{L_{c}}\times F\left(P_{\mathrm{ran}.}\right)\times G\times C^{1/4}$$

Therefore, the power of thermal convection takes the following form:

(10) $$P_{c}=h_{0}A\left(T-T_{s}\right)+h_{c}A{\left(T-T_{s}\right)}^{5/4}$$

### 2.2. Heat Equation of Conduction

The one-dimensional equation of heat conduction along the z direction without a heat source as a function of time can be written as [35]:

(11) $$\rho C\frac{\partial T}{\partial t}=k\frac{\partial^{2}T}{\partial z^{2}}$$

where ρ is the material density, C is the heat capacity of the material, t is the time, k is the thermal conductivity of the material, and z is the distance from the bottom sample surface. Equation (11) describes the dynamics of the temperature distribution over all of the time axes.

To fit the application for commercial LED heat sink, the steady state of the temperature distribution within the sample is a reliable parameter for estimating the heat flow of the material. Therefore, we can set Equation (11) into a steady state condition without a time dependence, as follows:

(12) $$0=k\frac{\partial^{2}T}{\partial z^{2}}\to \frac{\partial^{2}T}{\partial z^{2}}=0$$

The general solution of Equation (12) is:

(13) $$T\left(z\right)=c_{1}+c_{2}z$$

where c_1_, c_2_ are both constants of integration, determined by the boundary conditions (BCs) of temperature distribution, and T(z) is a linear function of z. The current aluminum sample with a ceramic coating on the top surface can be assumed as the gradation of three layers: pure aluminum, a mixture of aluminum and ceramic powders, and pure ceramics, as indicated in Figure 1a. Each layer has a thickness expressed by z_2_–z_1_ and L-z_2_. The input power $\dot{Q}$ of the power supply from the bottom surface is nearly constant during the working time of LED and in our thermal dissipation experiments. On reaching a steady state, the temperature and heat flow on both sides of an interface should be equal. Hence, we have the BCs:

(14) $$T\left(z_{1}^{-}\right)=T\left(z_{1}^{+}\right),\;T\left(z_{2}^{-}\right)=\;T\left(z_{2}^{+}\right)$$

(15) $$k_{1}\frac{\mathrm{dT}\left(z\right)}{\mathrm{dz}}{|}_{z=z_{1}^{-}}=k_{2}\frac{\mathrm{dT}\left(z\right)}{\mathrm{dz}}{|}_{z=z_{1}^{+}}$$

(16) $$k_{2}\frac{\mathrm{dT}\left(z\right)}{\mathrm{dz}}{|}_{z=z_{2}^{-}}=k_{3}\frac{\mathrm{dT}\left(z\right)}{\mathrm{dz}}{|}_{z=z_{2}^{+}}$$

where $k_{1}$, $k_{2}$, $k_{3}$ are the thermal conductivities of pure aluminum, the mixture of aluminum and ceramic, and pure ceramic. It is important to recall that heat loss occurs through thermal radiation and convection on the sample surface. Thus, the BC of the heat flow at the surface of the ceramic coating is determined by:

(17) $$-k_{3}\frac{\mathrm{dT}}{\mathrm{dz}}{|}_{z=L}=\sigma \varepsilon \left(T_{t}^{4}-T_{s}^{4}\right)+h_{c}{\left(T_{t}-T_{s}\right)}^{5/4}+h_{0}\left(T_{t}-T_{s}\right)$$

where L is the sample thickness and $T_{t}$ is the temperature of the sample top. For the sake of simplicity, the following parameters are denoted as:

(18) $$\dot{H}=\sigma \varepsilon \left(T_{s}^{4}-T_{t}^{4}\right)-h_{c}{\left(T_{t}-T_{s}\right)}^{\frac{5}{4}}-h_{0}\left(T_{t}-T_{s}\right),{\;k}_{12}=\frac{1}{k_{1}}-\frac{1}{k_{2}},k_{23}=\frac{1}{k_{2}}-\frac{1}{k_{3}}$$

The temperature distributions over these layers in terms of these parameters are:

(19) $$T\left(z\right)=\frac{\dot{H}}{k_{1}}z+T_{b},\;0\leq z<z_{1}$$

(20) $$T\left(z\right)=\frac{\dot{H}}{k_{2}}z+k_{12}{\dot{H}z}_{1}+T_{b},z_{1}\leq z<z_{2}$$

(21) $$T\left(z\right)=\frac{\dot{H}}{k_{3}}z+\left(k_{23}z_{2}+k_{12}z_{1}\right)\dot{H}+T_{b},z_{2}\leq z\leq L$$

where T_b_ is the temperature at the bottom of sample. Since the thickness L of our current sample is about 5 mm, and L >> (z_2_–z_1_) and (L-z_2_) (in our samples, L ~ 5 mm, (z_2_−z_1_) ~ 10 μm, and (L-z_2_) ~ 10 μm), all the temperatures of the sample surfaces can be taken as an average temperature, $T_{t}$, when estimating the heat flow of all the sample surfaces. In other words, the testing sample and cement resistor can be regarded as isothermal cuboids, which match the model assumption used in Ref. [30,31,32]. Therefore, $T_{t}$ can be determined by the equation of energy conservation:

(22) $${\dot{Q}}_{s}+\dot{H}A=0$$

(23) $${\sigma \varepsilon \mathrm{AT}}_{t}^{4}+h_{c}A{\left(T_{t}-T_{s}\right)}^{5/4}+h_{0}A\left(T_{t}-T_{s}\right)-\left({\sigma \varepsilon \mathrm{AT}}_{s}^{4}+{\dot{Q}}_{s}\right)=0$$

where ${\dot{Q}}_{s}$ is the input power to the samples. Since the top surface of the coated sample has a different total emissivity to that of aluminum, Equation (23) should be modified for the coated samples as:

(24) $$\sigma \left(\varepsilon_{t}A_{t}+\varepsilon_{l}A_{l}+\varepsilon_{b}A_{b}\right)T_{t}^{4}+h_{c}A{\left(T_{t}-T_{s}\right)}^{\frac{5}{4}}+h_{0}A\left(T_{t}-T_{s}\right)-\left[\sigma \left(\varepsilon_{t}A_{t}+\varepsilon_{l}A_{l}+\varepsilon_{b}A_{b}\right)T_{s}^{4}+{\dot{Q}}_{s}\right]=0$$

where A_t_, A_l_, and A_b_ are the sample area of the top, lateral, and bottom surface, respectively, and $\varepsilon_{t}$, $\varepsilon_{l}$, and $\varepsilon_{b}$ are the averaged sample total emissivity of the top, lateral, and bottom surface as a function of the coverage (cov) of coating materials on the sample top, respectively, $\varepsilon_{t}=\left(1-\mathrm{cov}\right)\times \varepsilon_{\mathrm{Al}}+\mathrm{cov}\times \varepsilon_{\mathrm{coat}}$ and $\varepsilon_{l}=\varepsilon_{b}=\varepsilon_{\mathrm{Al}}$, with 0≤cov≤100%. $\varepsilon_{\mathrm{Al}}$ and $\varepsilon_{\mathrm{coat}}$ are the values of the total emissivity of aluminum and the coating material. The ${\dot{Q}}_{s}$ can be calculated by the conservation of energy as:

(25) $${\dot{Q}}_{s}=\dot{Q}-P_{c,\mathrm{cem}.}-P_{r,\mathrm{cem}.}-P_{\mathrm{cir}.}$$

where $P_{\mathrm{cir}.}$ is the power loss of the electronic breadboard, and $P_{c,\mathrm{cem}.}$ and $P_{r,\mathrm{cem}.}$ are the power of thermal convection and thermal radiation for the cement resistor, respectively. Similarly, $P_{c,\mathrm{cem}.}$ and $P_{r,\mathrm{cem}.}$ can be expressed as:

(26) $$P_{c,\mathrm{cem}.}=h_{0,\mathrm{cem}.}A_{c}\left(T_{c}-T_{s}\right)+h_{c,\mathrm{cem}.}A_{c}{\left(T_{c}-T_{s}\right)}^{5/4}\;P_{r,\mathrm{cem}.}={\sigma \varepsilon }_{\mathrm{cem}.}A_{c}\left(T_{c}^{4}-T_{s}^{4}\right)$$

where $T_{c}$ is the temperature of the cement resistor, $A_{c}$ is the surface area of the cement resistor, $\varepsilon_{\mathrm{cem}.}$ is the total emissivity of the cement resistor, and $h_{0,\mathrm{cem}.}$ and $h_{c,\mathrm{cem}.}$ can be obtained by inserting the dimension of cement resistor, 22 × 10 × 9 mm^3^, into Equations (4)–(7) and (9) in Section 2.1. These parameter values, used in the analytic model, can be found in Table 1 and Table 2. Overall, the ceramic coating results in an average temperature difference, $\Delta T_{a}$, between the coated and uncoated samples as:

(27) $$\Delta T_{a}=T_{a}-T_{a}^{\prime }$$

where $T_{a}$ and $T_{a}^{\prime }$ are the theoretically predicted temperatures of the coated and uncoated samples, respectively. The theoretically predicted temperature difference can be compared with that measured experimentally, $\Delta T_{e}$, as:

(28) $$\Delta T_{e}=T_{e}-T_{e}^{\prime }$$

where $T_{e}$ and $T_{e}^{\prime }$ are the experimentally measured average temperatures of the coated and uncoated samples, respectively.

## 3. Experimental Materials and Procedures

The substrate material was a commercially pure Al alloy, AA1050, with a nominal chemical composition of 99.5 wt % Al with minor Si and Fe less than 0.5 wt %. The 1050 Al plate, 5 mm in thickness, was cut into 40 × 20 × 5 mm^3^ sections. A smooth surface finish was attained by polishing using the 1000-grade SiC paper. In parallel, a commercial heat sink with 13 fins, each measuring 8 mm in height, 50 mm in length, and 1 mm in thickness, was also applied for UMCA, to explore the thermal radiation effect.

The UMCA coating was conducted using 2 mm diameter SUJ2 bearing steel balls for plate samples and 1 mm in diameter for fin samples. Steel balls with a smooth surface and high hardness value (R_C_ scale of 62) were applied as the energy deliverer and were vibrated by a vibration generator with a fixed vibration frequency of 20 kHz at the bottom of the chamber. The vibration generator was powered by a piezoelectric motor [36]. The vibration amplitude A was fixed at 40 μm in this study. The ultrasonic mechanical coating treatment time was fixed at 2 min for every single coating operation.

**Figure 2:**
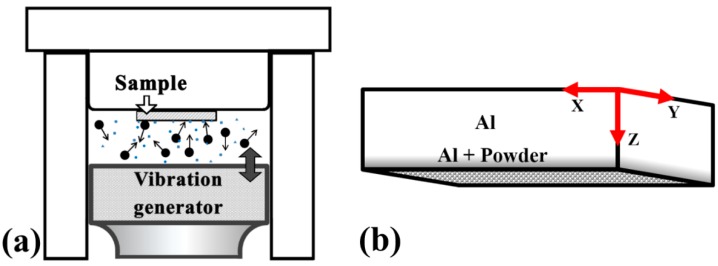
(**a**) Illustration of the setup and the finished sample of UMCA. The *X*, *Y*, and *Z* axes are defined in (**b**).

The pre-coating procedure was performed as follows. Approximately 1 gram of the SiO_2_ (termed as S), Al_2_O_3_ (termed as A), or graphite (termed as G) powders were added to ethanol (5 mL) and stirred into a suspension. The dimensions and total emissivity of these powders are summarized in Table 1. In comparison, it can be seen from Table 1, that graphite possesses the highest normal total emissivity of 0.98, followed by Al_2_O_3_ (0.94), SiO_2_ (0.9), and 1050 Al (0.11). In contrast, graphite has the highest thermal conductivity of about 470 W/mK along its 2D direction and about 25 W/mK along the vertical direction, followed by 1050 Al (235 W/mK), Al_2_O_3_ (12–38 W/mK), and SiO_2_ (1.3–1.5 W/mK). In some cases, different combinations using an equal weight of different powders were also tried, for example, applying SiO_2_ and Al_2_O_3_ (termed as SA) in a 1:1 weight ratio, or SiO_2_, Al_2_O_3_, and graphite in a 1:1:1 weight ratio (termed as SAG). A few drops of the suspension were placed on the surface and then spread out slowly to cover the entire substrate. After pre-coating, the substrates were dried in air and were then horizontally fastened above the chamber with the pre-coated surface facing downward, as shown in Figure 2a.

High-amplitude oscillations of the resonator resulted in an acceleration of the balls that initiated their chaotic motion inside the chamber and produced impacts on the pre-coated substrate surface. The pre-coating and ball impact treatments were repeated for different parameters. The duration of each impact treatment (termed as one time) was 2 min. Multiple times for such a treatment were tried and compared. After completing all of the coating treatments, the samples were rinsed with alcohol. 

The cross-sectional microstructures of the coatings were observed by using a JSM-6330F scanning electron microscope (SEM, JEOL, Tokyo, Japan), equipped with an energy dispersive spectroscope (EDS), in the secondary-electron or backscattered-electron mode, after the specimens were mounted, ground, and polished. The cross-sectional transmission electron microscopy (TEM) foils were fabricated using the dual-beam focused-ion-beam (FIB) system, with an operating voltage of 30 kV and an ion beam current of 1 pA. The TEM foils were examined by the Tecnai F20 field emission transmission electron microscope (FEI, Hillsboro, OR, USA), with an operating voltage of 200 kV.

**Figure 3:**
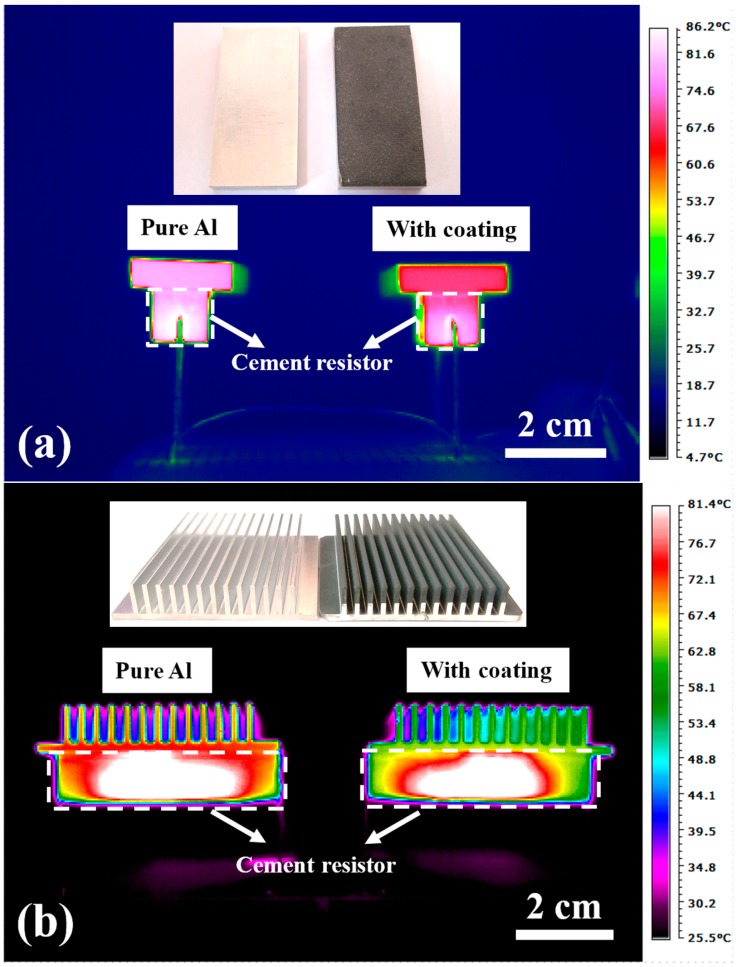
The setup of the thermal dissipation test under infrared imaging. The uncoated Al sample is shown on the left and the mechanically coated Al (this time with Al_2_O_3_ + SiO_2_) is on the right. The Al samples are placed above the cement resistor connected with the power supply: (**a**) plates; (**b**) fins.

To experimentally measure the temperature profile of the 5 mm Al substrate with and without ceramic plating, an EasIR-9 infrared thermal imaging camera (Guide, Wuhan, China) was used. The samples were placed on cement resistors and heated using electrical resistors, as demonstrated in Figure 1b. The input power and the temperature of the cement resistor were seriously calibrated before and after placing our samples. The temperature measurement was conducted in a constant-temperature room with a very low air flow and temperature deviation. We monitored the temperature of the testing environment and the system, and waited until it had completely reached a steady state. Additionally, the resulting temperature was measured by the infrared camera at the lateral side of the sample, where a small piece of reflective tape (Keyence op-91147) was used to reach the measuring accuracy. The usage of an infrared camera rather than thermal couples could limit the interference. The spectral range of the detector in an infrared camera is 8–14 μm, and the total emissivity of reflective tape in this range is 0.95. Before conducting the thermal dissipation test, all of the samples were ground to the same scale of 38 × 18 × 5 mm^3^. Therefore, the only coated surface which contributed to emissivity enhancement was the upper one, with an area of 38 × 18 mm^2^. We used a cement resistor as the heat source, and by controlling the input voltage, the generated heat energy could be managed. Different heat power levels were chosen, varying from 1–3 W. By extracting the temperature profile from the infrared camera, the effect of the coating could be evaluated and compared to the bare Al. Detailed setup of the thermal dissipation test under infrared imaging is shown in Figure 3.

## 4. Results and Discussion

### 4.1. Basic UMCA Morphology

**Figure 4:**
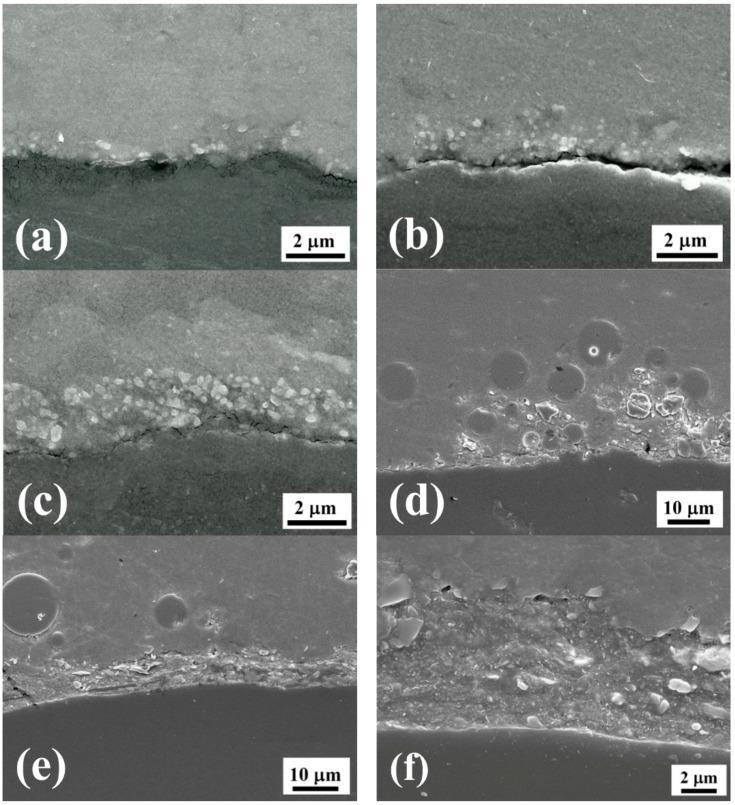
Typical cross-sectional SEM micrographs taken from samples: (**a**) 0.5 μm Al_2_O_3_ with 1 UMCA cycle; (**b**) 0.5 μm Al_2_O_3_ with 2 UMCA cycles; (**c**) 0.5 μm Al_2_O_3_ with 3 UMCA cycles; (**d**) mixed powders (15 μm Al_2_O_3_ + 15 μm SiO_2_) with 3 UMCA cycles; (**e**) mixed powders (15 μm Al_2_O_3_ + 15 μm SiO_2_ + graphite) with 3 UMCA cycles; and (**f**) enlarged micrograph of (**e**).

By the transfer of kinetic energy from the steel balls onto the pre-coated material, the ceramic powders were implanted into the target substrate. The particle size affects the inserted depth, which was, in our experiment, about two times that of the particle diameter for each UMCA run. For example, the ~0.5 μm Al_2_O_3_ particles would result in an inserted layer of about 1, 2, and 3 μm after 1, 2, and 3 UMCA cycles, as shown by the cross-sectional SEM micrographs in Figure 4a–c. At the bottom side, the Al_2_O_3_ particles occupy a very high volume fraction, and would gradually decrease in number with a deeper thickness. With increasing UMCA cycles, the inserted thickness continuously increases. 

In samples coated with micro-scaled (~15 μm in average) SiO_2_ or Al_2_O_3_ powders, the high energy bombardments would fracture some particles into some smaller ones (mostly about 5–10 μm). Such ~15 μm SiO_2_ or Al_2_O_3_ particles would produce an inserted layer of about 15, 25, and 40 μm after 1, 2, and 3 UMCA cycles, as shown by one representative cross-sectional SEM micrograph in Figure 4d. The micrograph for the mixture of three powders, (15 μm Al_2_O_3_ + 15 μm SiO_2_ + graphite), after three cycles, is presented in Figure 4e,f.

**Figure 5:**
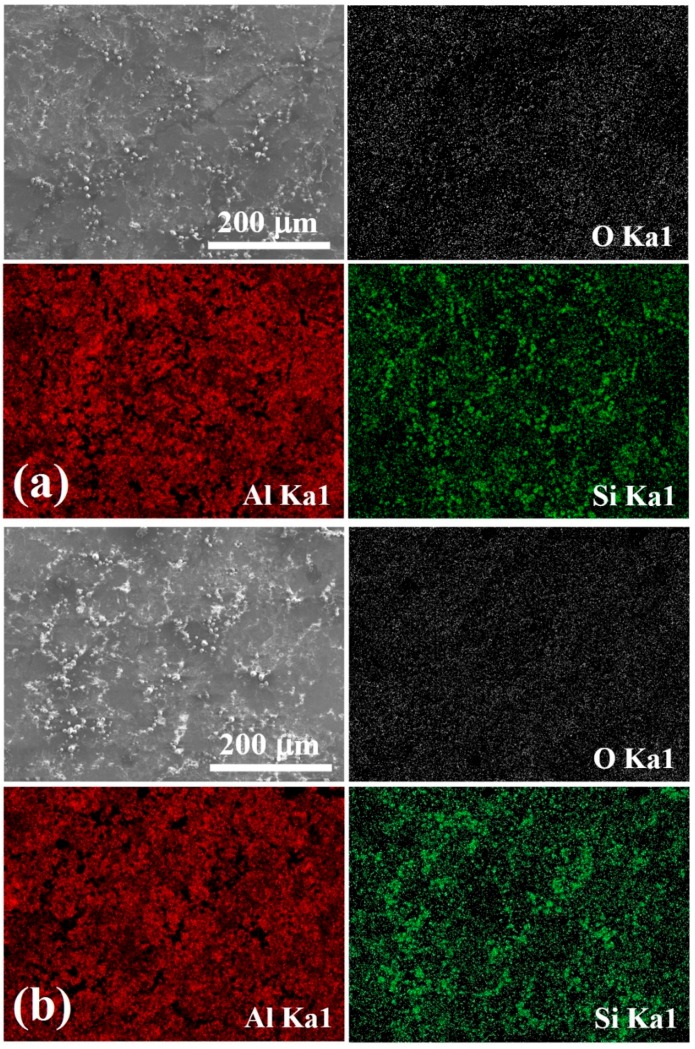

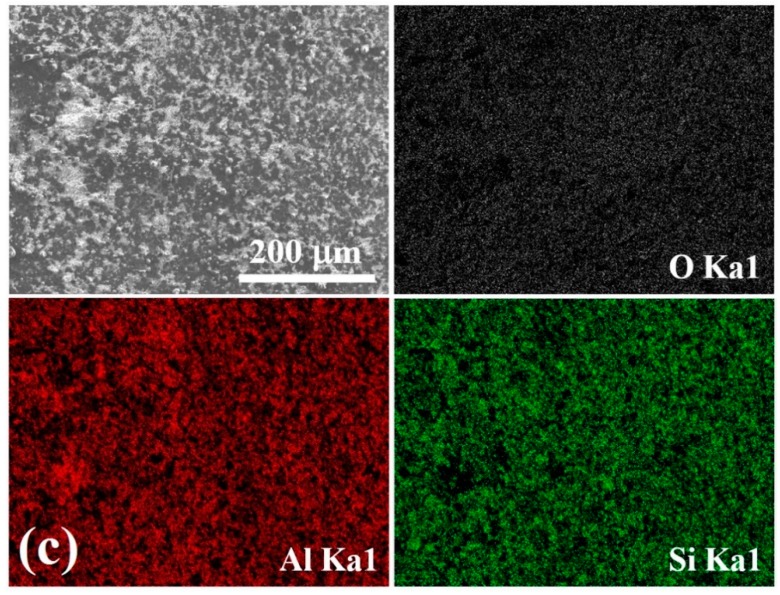
Typical SEM micrographs taken from the sample surface coated with 15 μm SiO_2_ powders for (**a**) 1 cycle; (**b**) 2 cycles; and (**c**) 3 cycles of UMCA, with the EDS mapping. The Si atoms occupy about from 30 to 60% area fraction of the whole analyzed area.

In the course of the theoretical model, the percentage of powder coverage on the surface is one of the parameters that needs to be inputted. Detailed multiple SEM images were taken for various cases using different powders and different UMCA cycles. Examples with 1, 2, and 3 UMCA cycles are shown in Figure 5, where the inserted powders can be defined and the coverage can be calculated by SEM/EDS mapping. The main focus of this research lies on the dissipation effect of the coatings, and the temperature drop is caused by the usage of the degrees of freedom for different coatings with high thermal emissivity. The coating materials, on the sample top, have a much higher total emissivity (~0.9) than that of Al bulk (~0.1). The averaged total emissivity of the sample top as a function of coverage was actually an effective quantity. Also, the coverage factor obtained by our analytical model is in accordance with that of the experimental results in Figure 5, obtained by EDS analysis from SEM, where the coverage factor can be obtained by image mapping software.

**Figure 6:**
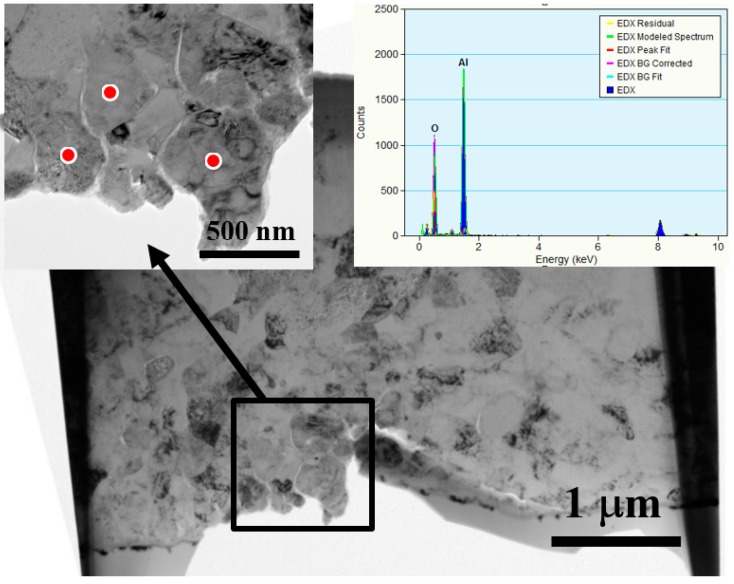
TEM images for the inserted 0.5 μm Al_2_O_3_ particles in the 1050 Al plate, with the EDS spectrum on the top right.

**Figure 7:**
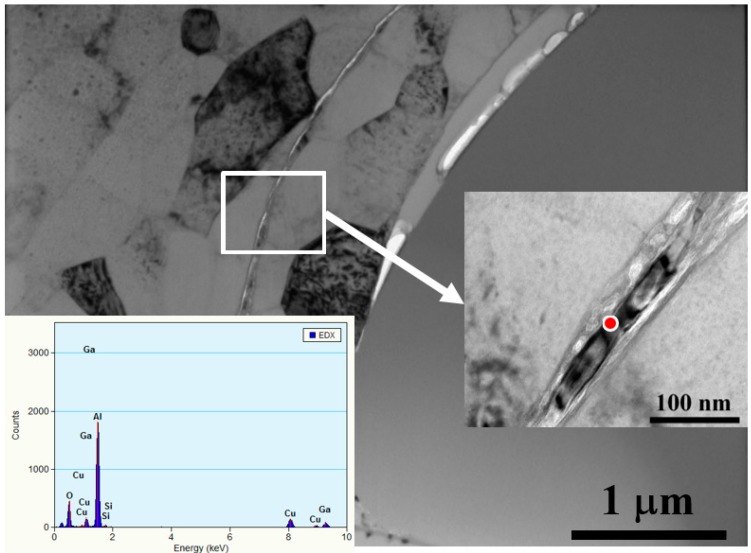
TEM images for the inserted ~15 μm SiO_2_ particle in the 1050 Al plate, with the EDS spectrum on the bottom left. Occasionally, there would be some gaps between the inserted SiO_2_ and Al matrix. Note that sometimes there is an external thin Al_2_O_3_ oxide layer (shown on the enlarged image on the right) inserted into the Al inner position with the mechanical coated large particles.

More detailed microstructures were revealed by TEM observations, as shown in Figure 6. The small sub-micron 0.5 μm Al_2_O_3_ particles are nicely and uniformly doped by UMCA into the Al matrix, with no apparent gap or second phase being generated. Sometimes, the external aluminum oxide thin layer can be bombarded into the Al inner matrix, inevitably forming interface gaps between the ceramic particles and Al matrix, as illustrated by the TEM micrograph in Figure 7. The gap is unwanted owing to its possibility of hindering thermal conduction. 

### 4.2. Effects of Surface Roughness on the Thermal Radiation

The surface roughness influences the emissivity. The emissivity with different roughness levels for Al alloys has been examined in several references [37,38]. Therefore, we have also conducted tests on the samples with only ball impact treatment, without adding powders. Compared to the as-ground sample, the temperature difference was 0.6 °C lower, indicating that the averaged emissivity of the sample top could have changed from 0.11 to 0.12, based on our analytical model. It can be seen that the roughness does impose some minor effects on emissivity enhancement in comparison with ceramic powder coatings, but this minor effect should not overshadow the major effect of the various ceramic powders.

### 4.3. Effects of Powder Size on the Thermal Radiation

The effect of ceramic powder size on the thermal radiation efficiency was examined for the uncoated and coated samples, with different sizes of powders. One example using Al_2_O_3_ powders of 0.5 and 15 μm in size is presented in Table 3. The coverage of the top surface by such Al_2_O_3_ powders is 34% and 55% for 0.5 and 15 μm, respectively. As a control group for comparison with the coated samples, the experimental top surface temperature, T_e_, for an uncoated 1050 Al plate was recorded. Then, the experimental top surface temperature for the coated samples, T_e_’, were measured. The temperature difference, ΔT_e_, is the term for the evaluation of the thermal radiation efficiency by the coated ceramic powders. From Table 3, it can be seen that ΔT_e_ for the 0.5 μm Al_2_O_3_ after 3 UMCA cycles is 3.6 ± 0.3 °C, and that for the 15 μm is 5.3 ± 0.2 °C. The theoretically predicted values are also compiled in Table 3. The calculated value of ΔT_a_ is 3.5 °C for 0.5 μm Al_2_O_3_ and 5.6 °C for 15 μm Al_2_O_3_. The value matches and the variation trends between the theoretical ΔT_a_ and experimental ΔT_a_ values are both good. When putting all the parameters in Table 1 and Table 2 into the power of thermal radiation and convection, the contributions from different modes of heat transfer can be individually calculated. We found that the ranking, P_c_ > P_r_ >> P_conduction_, where P_c_, P_r_, and P_conduction_ represented the power of thermal convection, radiation, and conduction, respectively. For example, in the cases with $\dot{Q}$ = 2.4 W, the rate of heat dissipation for bare Al from convection and radiation is about 92.3% and 7.7%, respectively, while the rate of heat dissipation for samples with an Al_2_O_3_ coverage of 55% is about 82.0% and 18.0%, respectively.

Since the larger powders would be more efficiently inserted into the Al plate at a much deeper position, as well as with a higher surface coverage, as demonstrated in Figure 4 and Table 3, the coated samples with larger powders could conduct more thermal radiation by these inserted powders, resulting in a lower top surface temperature T_e_’ and a higher ΔT_e_.

### 4.4. Effects of UMCA Cycles on the Thermal Radiation

As more UMCA cycles were conducted, more ceramic powders were spread on the surface and more powders were inserted into the inner depth, so the radiation efficiency of the coated sample surfaces was higher. Table 4 presents a comparison of the UMCA experiments using 15 μm Al_2_O_3_ powders for 1 to 3 cycles. The coverage of the top surface is 41%, 51%, and 55% for cycles 1 to 3, respectively. The experimental temperature drops with respect to the uncoated Al, and the ΔT_e_ value is 3.7 ± 0.2, 4.8 ± 0.3, and 5.3 ± 0.2 °C for cycles 1 to 3, respectively. In parallel, the calculated value of ΔT_a_ is 3.2, 5.2, and 5.6°C for cycles 1 to 3. The experimentally observed trend is highly consistent with the prediction. Similar results were obtained in 15 μm SiO_2_ powders cases, as listed in Table 5.

### 4.5. Effects of Heat Input Power on the Thermal Radiation

It is known that the thermal radiation efficiency is higher when there is a higher surface temperature, representing a higher heat input. Table 6 shows the case, using 15 μm Al_2_O_3_ powders for 3 cycles, where the heat input power source values used are 1.6 and 2.4 W. It can be seen that the experimental temperature drops with respect to the uncoated Al, and the ΔT_e_ value is 4.0 ± 0.1 and 5.3 ± 0.1 °C for 1.6 and 2.4 W, respectively. The experimental results are in perfect agreement with the theoretical prediction, ΔT_a_, which is 3.6 and 5.6 °C for 1.6 and 2.4 W, respectively.

### 4.6. Comparison of Thermal Radiation by Difference Powder Combinations

Based on the above systematic examinations of the effects of powder size, UMCA cycles, and heat input power, the subsequent experiments were conducted to examine the powder combinations in use.

First, a single type of powder, namely, either SiO_2_, Al_2_O_3_, or graphite, with a particle diameter of ~15 μm, coated on 1050 Al for three UMCA cycles, was connected to a heat input power source of 2.4 W. The experimental temperature drops with respect to the uncoated Al, and the ΔT_e_ values are listed in Table 7 for SiO_2_, Al_2_O_3_, and graphite. The ΔT_e_ values are 5.0 ± 0.2, 5.3 ± 0.2, and 6.3 ± 0.3 °C, respectively. The normal total emissivity, ε, for SiO_2_, Al_2_O_3_, and graphite, is 0.90, 0.94, and 0.98, respectively (Table 1). It is logical that with a higher infrared emissivity, the radiation efficiency can be higher, resulting in a higher temperature drop. Among these three types of powders, graphite behaves the best.

In early research, it was found that natural graphite has a layered and planar structure, composed of graphene units. There is only a weak van der Waals force between each layer, allowing layers of graphite to be easily separated by the severe bombarding of the steel balls during UMCA. This results in a flat and uniform coating to reach a nearly full coverage of the free surface. Therefore, the radiation efficiency of graphite is the highest.

Secondly, we attempted to apply the combination of two or three types of powders, namely, SiO_2_+Al_2_O_3_ (SA) and SiO_2_+Al_2_O_3_+Graphite (SAG), with a weight ratio of 1:1 and 1:1:1, respectively. Table 7 also lists the experimental temperature drops with respect to the uncoated Al, ΔT_e_, for SA and SAG for 3 and 10 cycles. The SA results with two kinds of spherical powders are similar to the case using only a single type of powder. However, the SAG results are more promising. The graphite thin sheets can be coated more effectively with the spherical SiO_2_ and Al_2_O_3_ powders, and they allow the planar graphite to be inserted into the deeper inner part of the sample. A better surface coverage and inserted depth are achieved when the three powders are mixed and coated together. The resulting experimental temperature drops with respect to the uncoated Al, and the ΔT_e_ value can reach 7.7 ± 0.3 and 8.2 ± 0.2 °C, respectively, for 3 and 10 UMCA cycles.

For mixed powders containing graphite, the coating morphology such as the consistency, surface continuity, and coverage is much better than those without graphite, as shown in Figure 4e,f. Using our SEM observations, for graphite-added cases, graphite plays the role of filling the gap between the hard particles of Al_2_O_3_ and SiO_2_ due to its easy fracture property, resulting in a higher coverage on the surface. This coverage can achieve a value as high as 95 ± 2%. The effective emissivity is thus higher than the cases in which graphite powders are not added. Another possibility for the improvement in thermal tests may be the high thermal conductivity of graphite (graphene), where the heat can be quickly transmitted and dispersed to the whole surface, enhancing both radiation and convection.

### 4.7. Application to Real Heat Sink Fins

With the more promising temperature drop by the combination of various powders, this scheme was thus applied to the commercial heat sink with 13 fins. In Equation (1), one can learn that the heat of radiation is related to the emissivity and the area of the surface. Because the movements of the steel balls during UMCA occur in three directions, the surface in the gap between the two fins can also be coated, achieving a high coverage of the total surface area. For the above Al plate samples, the coating was only conducted on one side of the sample. For the commercial fin samples, the coating can be applied to both fin sides. A higher radiation effect is expected.

The results are compiled in Table 7. Since we need to achieve a similar temperature range for the commercial fins and the experimental plate samples (the commercial fin samples have a much larger dimension), we need to input a higher power of 6.0 W to reach a similar surface temperature to the uncoated Al. The experimental temperature drop with respect to the uncoated Al, ΔT_e_, was further raised to 8.4 ± 0.2 and 11.1 ± 0.3 °C while using the SA and SAG powder mixtures, respectively. Note that the 11 °C temperature difference is considered to be a major achievement in industry. 

### 4.8. Comparison of the Analytic Model and Experimental Observations

All of the above experiment results are carefully compared with the analytical model, i.e., the theoretical prediction. The theoretically predicted values for various cases are also listed in Table 3, Table 4, Table 5 and Table 6, where all of the matches are satisfactory, including the increasing or decreasing trends and the exact values. The high agreement can also ensure that we can estimate the temperature drop for other samples of different shapes, or other coatings by different powder combinations. For example, the theoretically predicted temperature drop, ΔT_a_, for a plate Al sample coated with SAG for 10 cycles under a heat input power of 2.4 W, is 8.2 °C. The experimentally measured result, ΔT_e_, for SAG for 3 cycles is 7.7 °C, suggesting that the coverage of SAG powder on the top surface of the sample has almost reached the limitation.

In Table 3, Table 4, Table 5 and Table 6, there is a minor discrepancy between the predicted and experimental data. The model was constructed under the condition that all inserted particles are in perfect contact with the Al matrix. However, as illustrated in the SEM and TEM observations, there are inevitably some occasional gaps and discontinuous spots. Thus, the effective surface areas for thermal radiation may not be the same as is supposed in the model.

### 4.9. Closing Remarks

The heat sink does play an important role in LED and electronic devices. The design of heat sink affects the heat dissipation efficiency [39,40]. Without a good thermal dissipating path, heat may accumulate and may cause a temperature rise in the LED chip. In previous researches [11], the results showed that the life time and the light output of the LED drops dramatically with an increase of temperature. The traditional solution for dissipation in high power LEDs is to use a fan as forced convection air cooling. However, there are many disadvantages and drawbacks in this manner, such as the requirement for extra energy to power the motor, and the decline of the system in a moist and dusty environment, etc. Our current UMCA surface composite layer provides a more functional and practical way of dissipating heat, by simply adding an additional path of radiation. The present sample with only one face coating can reach an appreciable upgrade. Heat sink materials can be fabricated using thin sheets with fins, coated on both sides, to accomplish an even better efficiency of heat dissipation, which is also conductive to temperature decrement in a high-power device like GPU, where an extra cooling system is necessary. According to previous research [41], a 5 °C reduction can increase the LED life time by 10,000 h. Our design is a promising potential candidate for replacing Al as a heat sink material.

## 5. Conclusions

In this work, the UMCA technique was applied on Al plate samples and fin samples coated with various ceramic materials, such as SiO_2_, Al_2_O_3_, and graphite. The following conclusions can be drawn:

(1) The coated ceramic powder size imposes an effect on the resulting thermal radiation efficiency. The large ~15 μm powders perform better than the small ~0.5 μm powders.
(2) With an increasing number of UMCA cycles, the powders can be spread over a higher surface area and can be inserted into deeper locations. Both effects result in a higher thermal radiation efficiency.
(3) For the same coating, the thermal radiation efficiency is higher under a higher input heat power, namely, a higher thermal radiation efficiency occurs at higher temperatures.
(4) In comparison with the three powders in use, graphite (with a total emissivity of 0.98) performs the best, followed by Al_2_O_3_ (0.94) and SiO_2_ (0.90).
(5) A mixture of different powders, such as SiO_2_+Al_2_O_3_+Graphite, results in an even better thermal radiation efficiency. For the 1050 Al plate sample with only one surface side coated by UMCA, the temperature drop with respect to uncoated Al was 8.2 °C.
(6) For commercial heat sink samples with multiple fins, UMCA can be conducted on both sides of the fins. The resulting temperature drop with respect to uncoated Al was as high as 11.1 °C.
(7) An analytical model was established, and the theoretically predicted temperature drop with respect to Al is a prescreening evaluation for heat sink samples of different shapes, different dimensions, coatings of different ceramic powders, and different amounts of coating. Overall, the agreements between the model predictions and experimental measurements are satisfactory.

## Figures and Tables

**Table 1:** Summary of the diameters and emissivity ε of the powders. The base of 1050 Al has an emissivity ε of 0.11 and a heat conductivity k of 235 W/mK.

Powder	SiO_2_	Al_2_O_3_ (1)	Al_2_O_3_ (2)	Graphite
Diameter (μm)	~15	~0.5	~15	~15
Normal total emissivity, ε	0.9	0.94	0.94	0.98
Thermal conductivity, k (W/mK)	1.3–1.5	12–38	12–38	25–470

**Table 2:** Summary of the convection coefficient used in the analytic model. During the thermal dissipation tests, the surrounding temperature $T_{s}$ was fixed at 297 K, $L_{c}$ of the testing samples; the cement resistors were $4.13\times 10^{-2}$ m and $2.82\times 10^{-2}$ m, respectively; the C values of the testing samples and cement resistors were $8.26\times 10^{3}{\;K}^{-1}$ and $2.63\times 10^{3}\;K^{-1}$, respectively; and the $R_{a}$ values were between $10^{5}$ and $10^{6}$.

Convection Coefficient	$h_{0}$	$h_{c}$	$h_{0,cem.}$	$h_{c,cem.}$
Value	2.37 W/m^2^K	2.92 W/m^2^K^1.25^	3.21 W/m^2^K	3.43 W/m^2^K^1.25^

**Table 3:** Effects of powder size when comparing the temperatures obtained from the analytical model (all with subscript a) and the experiment (all with subscript e), using two Al_2_O_3_ powder sizes (0.5 and 15 μm), both for three UMCA cycles and both under the total heat input power $\dot{Q}$ of 2.4 W. The coverage of the top surface is 34% and 55% for 0.5 and 15 μm, respectively.

Al_2_O_3_ Size (μm)	UMCA Cycle	$\dot{Q}$ (W)	T (°C)		T’ (°C)		ΔT (°C)	
			T_a_	T_e_	T_a_’	T_e_’	ΔT_a_	ΔT_e_
0.5	3	2.4	83.9	83.5 ± 0.3	80.4	79.9 ± 0.4	3.5	3.6 ± 0.3
15	3	2.4	83.9	83.5 ± 0.3	78.3	78.2 ± 0.2	5.6	5.3 ± 0.2

**Table 4:** Effects of UMCA cycles when comparing the temperatures obtained from the analytical model (all with subscript a) and the experiment (all with subscript e), using the 15 μm Al_2_O_3_ powders for one to three UMCA cycles and all under a total heat input power $\dot{Q}$ of 2.4 W. The coverage of the top surface is 41%, 51%, and 55% for cycles 1–3, respectively.

Al_2_O_3_ Size (μm)	UMCA Cycle	$\dot{Q}$ (W)	T (°C)		T’ (°C)		ΔT (°C)	
			T_a_	T_e_	T_a_’	T_e_’	ΔT_a_	ΔT_e_
15	**1**	2.4	83.9	83.5 ± 0.3	79.7	79.8 ± 0.2	3.2	3.7 ± 0.2
15	**2**	2.4	83.9	83.5 ± 0.3	78.7	78.7 ± 0.3	5.2	4.8 ± 0.3
15	**3**	2.4	83.9	83.5 ± 0.3	78.3	78.2 ± 0.2	5.6	5.3 ± 0.2

**Table 5:** Effects of UMCA cycles when comparing the temperatures obtained from the analytical model (all with subscript a) and the experiment (all with subscript e), using the 15 μm SiO_2_ powders for one to three UMCA cycles and all under a total heat input power $\dot{Q}$ of 2.4 W. The coverage of the top surface is 34%, 54%, and 58% for cycles 1–3, respectively.

SiO_2_ Size (μm)	UMCA Cycle	$\dot{Q}$ (W)	T (°C)		T’ (°C)		ΔT (°C)	
			T_a_	T_e_	T_a_’	T_e_’	ΔT_a_	ΔT_e_
15	**1**	2.4	83.9	83.5 ± 0.3	80.5	80.3 ± 0.2	3.4	3.2 ± 0.2
15	**2**	2.4	83.9	83.5 ± 0.3	78.7	78.9 ± 0.3	5.2	4.6 ± 0.3
15	**3**	2.4	83.9	83.5 ± 0.3	78.3	78.5 ± 0.2	5.6	5.0 ± 0.2

**Table 6:** Effects of the heat input power when comparing the temperatures obtained from the analytical model (all with subscript a) and the experiment (all with subscript e), using the 15 μm Al_2_O_3_ powders for three UMCA cycles and under two total heat input power $\dot{Q}$ levels.

Al_2_O_3_ Size (μm)	UMCA Cycle	$\dot{Q}$ (W)	T (°C)		T’ (°C)		ΔT (°C)	
			T_a_	T_e_	T_a_’	T_e_’	ΔT_a_	ΔT_e_
15	3	**1.6**	65.7	66.2 ± 0.1	62.1	62.2 ± 0.1	3.6	4.0 ± 0.1
15	3	**2.4**	83.9	83.5 ± 0.3	78.3	78.2 ± 0.2	5.6	5.3 ± 0.2

**Table 7:** Summary of dissipating effects for the coatings of different powders, all of a ~15 μm size and all under the same heat input power $\dot{Q}$ of 2.4 W for the simple plate and 6.0 W for the complicate-shaped fin.

Coating	Powder Size (μm)	Cycle	$\dot{Q}$ (W)	T (°C)	T’ (°C)	ΔT (°C)
**Coating on the single plate**						
SiO_2_	~15	3	2.4	83.5 ± 0.3	78.5 ± 0.2	5.0 ± 0.2
Al_2_O_3_	~15	3	2.4	83.5 ± 0.3	78.2 ± 0.2	5.3 ± 0.2
Graphite	~15	3	2.4	83.5 ± 0.3	77.2 ± 0.3	6.3 ± 0.3
SiO_2_+Al_2_O_3_	~15	3	2.4	83.5 ± 0.3	78.9 ± 0.1	4.6 ± 0.2
SiO_2_+Al_2_O_3_	~15	10	2.4	83.5 ± 0.3	76.9 ±0.3	6.6 ± 0.3
SiO_2_+Al_2_O_3_+Graphite	~15	3	2.4	83.5 ± 0.3	75.8 ± 0.3	7.7 ± 0.3
SiO_2_+Al_2_O_3_+Graphite	~15	10	2.4	83.5 ± 0.3	75.3 ± 0.1	8.2 ± 0.2
**Coating on a fin**						
Al_2_O_3_+SiO_2_	~15	3	6.0	88.7 ± 0.2	80.3 ± 0.2	8.4 ± 0.2
Al_2_O_3_+SiO_2_+Graphite	~15	3	6.0	88.7 ± 0.2	77.6 ± 0.3	11.1 ± 0.3
